# Smear positive pulmonary tuberculosis and associated risk factors among tuberculosis suspects attending spiritual holy water sites in Northwest Ethiopia

**DOI:** 10.1186/s12879-017-2211-5

**Published:** 2017-01-26

**Authors:** Dejene Derseh, Feleke Moges, Belay Tessema

**Affiliations:** 1 Department of Biomedical Sciences, College of Health Sciences, Mizan Tepi University, Mizan Teferi, Ethiopia; 2 Department of Medical Microbiology, College of Medicine and Health Sciences, University of Gondar, P.O. BoX 196, Gondar, Ethiopia

**Keywords:** Pulmonary tuberculosis, Holy water, Risk factors

## Abstract

**Background:**

Tuberculosis (TB) remains one of the world’s deadliest communicable diseases. In Ethiopia, tuberculosis patients have different pattern of health care seeking behavior. They usually adopt other approaches like traditional healers and spiritual holy water sites before consulting public health facilities. This study was aimed to assess the prevalence of smear positive pulmonary tuberculosis and associated risk factors among tuberculosis suspects attending spiritual holy water sites.

**Methods:**

A cross-sectional study was conducted from February 01, 2015 to March 30, 2015 in seven selected holy water sites in Northwest Ethiopia. During the study period, a total of 1384 adult holy water users were screened for PTB symptoms. A total of 382 pulmonary tuberculosis suspects participated in the study. Socio-demographic data were collected using a semi-structured questionnaire. Spot-morning-spot sputum specimens were collected and examined for acid fast bacilli using Auramine O fluorescence staining technique. Smear positive sputum samples were tested by GeneXpert MTB/RIF assay for rifampicin resistance. Descriptive statistics, binary and multivariate logistic regression analysis were employed using SPSS-16 software.

**Results:**

The prevalence of smear positive pulmonary tuberculosis was 2.9% with point prevalence of 795/100, 000 holy water users. History of contact with tuberculosis patient (AOR = 9.174, 95% C.I = 2.195–38.34) and the number of family members > 5 per household (AOR = 9.258, 95% C.I = 1.14–74.97) were significantly associated with smear positive pulmonary tuberculosis. Rifampicin resistance was not detected from all smear positives by GeneXpert MTB/RIF assay.

**Conclusions:**

The prevalence of smear positive pulmonary tuberculosis in spiritual holy water sites was 7.4 fold higher than the general population. History of contact with active tuberculosis patients and increased family size were significantly associated with smear positive pulmonary TB. The national tuberculosis program should consider spiritual holy water sites as potential foci for TB transmission and plan regular survey and health education in holy water sites for effective TB prevention and control in the country.

## Background

Tuberculosis (TB) remains one of the world’s deadliest communicable diseases [1]. Globally, the burden of TB is increasing at alarming rate and continues to be a major public health problem throughout the world with increasing incidences in developing countries. Tuberculosis is ranked as the second leading cause of death from an infectious disease worldwide, after human immunodeficiency virus (HIV) [2]. According to World Health Organization (WHO) global 2014 TB report, an estimated 9.0 million people develop TB and 1.5 million died from the disease in 2013 worldwide. Out of this more than half (56%) were in South East Asia and western pacific regions. African region accounts one quarter which had the highest rates of cases and deaths relative to the population [1, 3].

Despite the availability of efficacious anti-TB treatments for decades, TB remains a major global health problem [3]. The emergence of multidrug resistance tuberculosis (MDR-TB) and the pandemic of human immunodeficiency virus/acquired immunodeficiency syndrome (HIV/AIDS) worsen the burden of TB in Africa including Ethiopia [4, 5]. In Ethiopia, TB is a major cause of morbidity and mortality [6]. Ethiopia ranked 7th among the 22 high burden countries where 80% of total TB cases reported [7]. According to WHO 2014 report, the burden of TB in Ethiopia was 224, 211 and 32 per 100 000 population for new smear positive cases, prevalence and mortality, respectively [1]. In developing countries, the burden of TB is exacerbated by different conditions such as malnutrition, HIV/AIDS and also dependence on traditional healers which results in treatment delays [6, 8, 9].

In Ethiopia, many TB cases in the community are undiagnosed [10, 11]. The case detection rate of smear positive pulmonary TB is low compared with the 70% target by WHO [6]. It is indicated that, one of the underlying reason is that the high incidence countries like Ethiopia rely on the passive case finding strategy, which presupposes self presentation and willingness of TB suspects to the public health care facilities [12, 13]. But, studies showed that in Africa including Ethiopia, people with TB symptoms (TB suspects) first adopt other approaches before consulting public health facilities. Patients with the symptom of TB have different patterns of health seeking behavior and the period when to seek health is related with delay in diagnosis and treatment of tuberculosis [8, 14–17].

Tuberculosis patients initially visit informal health care providers prior to their first consultation to the public health facility. They prefer to seek treatment from a variety of sites which includes spiritual holy water sites (24%), private practitioners (13%), rural drug vendors (7%) and traditional healers (3%). Dependence on informal health care providers results delay in diagnosis and initiation of anti-TB treatment at public health care institutions. Pulmonary tuberculosis (PTB) patients delay seeking care at public health facilities while getting treatment from informal sources [8]. This increases the likelihood of developing MDR-TB and progression of disease which results increased mortality and enhanced TB transmission in the community [18].

In Ethiopia, holy water is used as an alternative treatment. Even though there are no available documented data to show the proportion of people that use holy water as their treatment option, it is a public knowledge that many Ethiopians use it as an alternative treatment for various diseases. Specifically, people with cough symptoms or other pulmonary TB suspects visit traditional healers and holy water sites as a source of care for their illness [8, 14]. Mostly, patients stay close to holy water sites for an extended duration of time until they are perceived to be cured.

Holy water sites are found at designated places with springs that are believed to have a power to cure individuals from various diseases. In this setting, a number of people who seek holy water treatment for their illness come together from various localities, leaving their homes and stay close together for certain period of time in houses built around holy water sites. These houses are small, crowded and poorly ventilated which increase the risk of TB transmission.

Most reports of TB were from public health care institutions but a high prevalence of TB was found by few community surveys. A number of TB patents (TB suspects) first visit informal health care providers rather than reporting to public health facilities. This threatens transmission of TB in the community and makes the passive case detection rate low. In Northwest Ethiopia, two-thirds of pulmonary TB in the community were undiagnosed and the prevalence of new sputum smear positive TB in the community was 174 per 100, 000 populations [10].

Even though studies indicate that pulmonary TB suspects and TB patients visit traditional healers and spiritual holy water sites as their treatment option, the prevalence of TB in spiritual holy water sites in Northwest Ethiopia has not been reported to date. Therefore, this study was conducted to estimate the prevalence and associated risk factors of smear positive pulmonary tuberculosis among those people who use spiritual holy water sites from February 01, 2015 to March 30, 2015.

## Methods

### Study area, study design and participants

A cross-sectional study was conducted in 7 selected holy water sites found in North Gondar zone: two holy water sites in Gondar town, two in Chilga district, two in Dembia woreda and one in Tikele Dingay. North Gondar zone is located 739 km away from the capital city, Addis Ababa towards the Northwest part of Ethiopia. Selected holy water sites are among popularly believed sites for holy water treatment which can accommodate large numbers of people and the high number of holy water users that travel to these sites every year. Northwest part of the country contains many holy water sites and high proportion of holy water users. In North Gondar zone, northwest part of Ethiopia, 95% of the population practice Ethiopian Orthodox Christianity which widely practice a faith based therapy with holy water [19].

The study was conducted from February 01, 2015 to March 30, 2015. The minimum sample size (n) was determined by using single population proportion formula [*n* = (Z α/2) ^2^ P (1-P) /d^2^], where Z_α/2_ = the value under standard normal table at 95% level of confidence which is 1.96, expected prevalence P, set at 50% to yield maximum sample size, d = precision which was set at 5%. Including 10% non response rate, the final sample size was 422 PTB suspects. However, a total of 382 PTB suspects participated in the study.

### Data collection and laboratory methods

Holy water users ≥ 15 years of age were interviewed using a questionnaire to identify pulmonary TB suspects. According to the national TB manual, individuals with persistent cough for two weeks or more and presence of other symptoms i.e. expectoration, blood contained sputum, fever, chest pain, shortness of breath, fatigue, night sweats, loss of appetite, loss of body weight and contact history with active TB patients were considered as suspects for PTB [6]. Socio-demographic characteristics included age, gender, occupation, residence, marital status, monthly income, family size and educational status. Other potential risk factors for TB included previous history of TB, history of contact with patients with active TB or chronic cough; history of previous stay at holy water sites; duration of stay at holy water sites; and sharing a room at these sites. Completeness of questionnaire was checked and three consecutive sputum samples (spot-morning-spot) were collected from each PTB suspects using clean, dry and leak-proof sputum cups. Sputum samples were immediately placed in a cold box and transported to Gondar University Hospital for laboratory processing. The samples were processed by Auramine O staining, fluorescent microscopy for acid fast bacilli (AFB) examination [20]. Smear positive samples were stored at -20°C until used and processed by GeneXpert MTB/RIF assay for Rifampicin (RIF) resistance testing [21–23].

### Data analysis and interpretation

Data were entered by using Epi info version 7.0 and transferred in to SPSS version 16 for cleaning, categorization and analysis. The results were summarized using descriptive statistics including frequencies and proportions. Bivariate analysis was conducted to identify the association between each independent variable with the outcome variables. Multivariate analysis was employed to identify independent predictors associated with the outcome variables. In the Multivariate analysis, adjusted odds ratio (AOR) and corresponding 95% confidence intervals were retrieved. Those variables with *P* value of less than 0.05 were considered as statistically significant.

## Results

### Socio demographic characteristics of respondents

Of the total 1384 holy water users, 382 (27.6%) individuals were identified as TB suspects. Among the pulmonary tuberculosis suspects, 253 (66.2%) were male while 129 (33.8%) were female. The mean (SD) age of study participants was 39 (17.2) years. More than half, 235 (61.5%) of respondents were married. Two hundred eighty (73.3%) participants were rural residents while 51.3% were farmers. Two hundred (52.4%) study participants had greater than 5 family members per household [Table 1].

**Table 1:** Socio-demographic characteristics of pulmonary tuberculosis suspects (*N* = 382)

Characteristics		Frequency (%)
Gender	Male	253 (66.2)
	Female	129 (33.8)
Age	15-30	126 (33.0)
	31-45	136 (35.6)
	≥46	120 (31.4)
Residence	Rural	280 (73.3)
	Urban	102 (26.7)
Marital status	Single	100 (26.2)
	Married	235 (61.5)
	Divorced	13 (3.4)
	Widowed	34 (8.9)
Education status	Can’t read and write	177 (46.3)
	Primary school	150 (39.3)
	High school (9-12)	47 (12.3)
	>12 grade	8 (2.1)
Total family size	1- 5	182 (47.6)
	>5	200 (52.4)
Occupation	Farmer	196 (51.3)
	House wife	57 (14.9)
	Employee	44 (11.5)
	Student	44 (11.3)
	Others^a^	41 (10.7)
Total monthly income	<500 ETB	246 (64.4)
	500-1000 ETB	79 (20.7)
	>1000 ETB	57 (14.9)

^a^Religious leaders, daily laborers, merchant, non-employed ETB = Ethiopian Birr

### Prevalence of smear positive pulmonary tuberculosis

Among 382 study subjects, 11 (2.9%) were smear positive for acid fast bacilli by Auramine O fluorescence staining technique. The point prevalence of smear positive PTB was 795/100,000 holy water users. Of total positives, 9/11 (81.8%) and 2/11 (18.2%) were males and females, respectively. The majority of smear positives, 8/11 (72.7%) were rural dwellers. Seven smear positives (63.6%) were married. A high proportion of smear positive pulmonary tuberculosis cases were reported among those > = 46 years old, 7/11 (63.6%) and among those respondents who had greater than 5 family members per household, 10/11 (90.9%). In addition, 7/11 (63.6%) of smear positives were farmers. However, none of smear positive cases were resistant for rifampicin [Table 2].

**Table 2:** Proportion of smear positive pulmonary tuberculosis with socio-demographic characteristics of study subjects

Variables		Prevalence of smear positive PTB	
		Positive No (%)	Negative No (%)
Gender	Male	9 (81.8)	244 (65.8)
	Female	2 (18.2)	127 (34.2)
Age groups	15-30	1 (9.1)	125 (33.7)
	31 -45	3 (27.3)	133 (35.8)
	≥46	7 (63.6)	113 (30.5)
Residence	Rural	8 (72.7)	272 (73.7)
	Urban	3 (27.3)	99 (26.3)
Marital status	Single	2 (18.2)	98 (26.4)
	Married	7 (63.6)	228 (61.5)
	Divorced	1 (9.1)	12 (3.2)
	Widowed	1 (9.1)	33 (8.9)
Education status	Can’t read and write	6 (54.5)	171 (46.1)
	Primary school	3 (27.3)	147 (39.6)
	High school (9-12)	1 (9.1)	46 (12.4)
	>12 grade	1 (9.1)	7 (87.5)
Total family size	1 -5	1 (9.1)	181 (48.8)
	>5	10 (90.9)	190 (51.2)
Occupation	Farmer	7 (63.6)	189 (50.9)
	House wife	1 (9.1)	56 (15.1)
	Employed	1 (9.1)	43 (11.6)
	Student	1 (9.1)	43 (11.6)
	Others^a^	1 (9.1)	40 (10.8)
Total monthly income	<500 ETB	9 (81.8)	237 (63.1)
	500 -1000 ETB	1 (9.1)	78 (21.0)
	>1000 ETB	1 (9.1)	56 (15.1)
Total		11 (2.9)	371 (97.1)

^a^Religious leaders, daily laborers, merchant, non-employed, *ETB = *Ethiopian birr

### Risk factors for smear positive pulmonary tuberculosis

Based on bivariate logistic regression, family size was significantly associated with smear positive pulmonary tuberculosis (COR = 9.526, 95% C.I = 1.207-75.169) [Table 3].

**Table 3:** Bivariate analysis of socio-demographic variables of study subjects associated with smear positive pulmonary tuberculosis

Variables		Prevalence of smear positive PTB			*p*-value
		Positive No	Negative No	COR (95% C.I)	
Gender	Male	9	244	2.432 (0.499-11.003)	0.281
	Female	2	127	1	
Age groups	15-30	1	125	1	0.372
	31 -45	3	133	2.280 (0.289-27.464)	
	≥46	7	113	7.743 (0.938 -63.912)	0.057
Residence	Rural	8	272	0.971 (0.252-3.732)	0.965
	Urban	3	99	1	
Marital status	Single	2	98	1	0.444
	Divorced/Widowed	2	45	2.178 (0.297-15.955)	
	Married	7	228	1.504 (0.307-7.371)	0. 614
Education status	Can’t read and write	6	171	0.246 (0.026-2.325)	0.221
	Primary school	3	147	0.143 (0.013-1.554)	0.110
	High school (9-12)	1	46	0.152 (0.009-2.721)	0.201
	>12 grade	1	7	1	
Family size	1 -5	1	181	1	0.032
	>5	10	190	9.526 (1.207-75.169)	
Occupation	Farmer	7	189	1.481 (0.177-12.378)	0.717
	House wife	1	56	0.714 (0.043-11.762)	0.814
	Employed	1	43	0.930 (0.056-15.375)	0.960
	Student	1	43	0.930 (0.056-15.375)	0.960
	Others^a^	1	40	1	
Total monthly income	<500 ETB	9	237	2.127 (0.264-17.132)	0.478
	500 -1000 ETB	1	78	0.718 (0.044-11.724)	0.816
	>1000 ETB	1	56	1	

^a^Religious leaders, daily laborers, merchant, non-employed, *ETB = *Ethiopian birr, No = number

Similarly, history of contact with active TB patient was significantly associated with smear positive pulmonary tuberculosis (COR = 5.649, 95% C.I = 1.673 - 19.071). However, other factors such as history of previous TB, knowledge about TB, history of previous stay at holy water sites and duration of days spent at holy water sites were not significantly associated with smear positive pulmonary tuberculosis [Table 4].

**Table 4:** Bivariate analysis of risk factors for smear positive pulmonary tuberculosis among study subjects

Variables	Positive *N*	Negative *N*	COR (95% CI)	*p*- value
History of contact with chronic coughers				0.181
Yes	6	128	2.278 (0.682-7.609)	
No	5	243	1^a^	
History of contact with TB patients				0.005
Yes	6	65	5.649 (1.673-19.071)	
No	5	306	1^a^	
Heard about PTB disease				0.121
Yes	9	349	1^a^	
No	2	22	3.525 (0.718-17.315)	
Know PTB is transmittable disease				0.835
Yes	8	280	1^a^	
No	3	91	1.154 (0.300-4.441)	
Had PTB disease before				0. 351
Yes	1	13	2.754 (0.328-23.146)	
No	10	358	1^a^	
History of previous stay at holy water sites				0.212
Yes	7	164	2.209 (0.636-7.675)	
No	4	207	1^a^	
No of days spent at HWS				0.987
1-17 days	8	269	1.011 (0.263-3.886)	
> 17 days	3	102	1^a^	
Sharing containers for drinking at HWS				0.093
Yes	9	202	3.765 (0.802-17.663)	
No	2	169	1^a^	
Share room at HWS				
Yes	10	252	4.722 (0.598-37.316)	0.141
No	1	119	1^a^	

^a^Reference category, *COR = *crude odds ratio, *95% CI = *95% confidence interval, *HWS = *Holy water site

After multivariate logistic regression analysis, family size (*p* = 0.037) and history of contact with tuberculosis patient (*p* = 0.002) were significantly associated with smear positive pulmonary tuberculosis. Respondents who have greater than five family members per household were about nine times (AOR = 9.258, 95% C.I = 1.14–74.97) more likely to develop smear positive pulmonary tuberculosis compared to those with < 5 family members per household. With respect to contact history, those respondents who had previous history of contact with tuberculosis patient were about nine times (AOR = 9.174, 95% C.I = 2.195–38.34) more likely to develop smear positive pulmonary tuberculosis than those who had no history of contact [Table 5].

**Table 5:** Multivariate analysis of risk factors for smear positive pulmonary tuberculosis among study subjects

Variables	Positive No	Negative No	COR (95% CI)	*P*- value	AOR (95% CI)	*P*- value
Familysize						
1-5	1	181	1^a^		1^a^	0.037
> 5	10	190	9.52 (1.207-75.169)	0.032	9.258 (1.14-74.97)	
History of contact with TB patients						
Yes	6	65	5.64 (1.673-19.071)	0.005	9.174 (2.195- 38.34)	0.002
No	5	306	1^a^		1^a^	

*AOR = *adjusted odds ratio, *95% CI = *95% confidence interval, ^a^Reference category

## Discussion

This study provide insights on the prevalence of smear positive pulmonary tuberculosis and point out possible risk factors associated with smear positive PTB among holy water users. The point prevalence of 795/100, 000 holy water users observed in this study was 7.4 times higher than the results of Ethiopian national population based TB prevalence survey [24]. This difference might be due to the difference in the study population in which the national TB survey was conducted which excluded congregate settings and high risky areas like monasteries and holy water sites. The higher rate of smear positive cases at holy water sites could be due to overcrowding, close contact and poorly ventilated conditions which favor the high rate of bacilli transmission since a number of holy water users stayed together for an extended duration. In addition, it previously was noted that TB suspects and TB patients visit traditional healer sites like holy water sites as their treatment option before reaching to the public health facilities [8, 14]. Therefore, the high rate of smear positive cases detected at holy water sites could be due to the high number of TB suspects visiting holy water sites as their treatment option. Most people who attend holy water sites prefer to manage various diseases, including TB spiritually and consider spiritual holy water sites as their preferred treatment option [25]. In Ethiopia, traditional medicines including holy water sites are used by 80% of the population [26]. Most people from rural residences and older age groups prefer to consult spiritual sites like holy water for their illness rather than public health care institutions due to different reasons such as lack of awareness, long distance of public health care facilities and socio-economic factors [8].

The result of this study is similar to the result of a study conducted in rural district of southern Ethiopia which reported 3% smear positive PTB among PTB suspects [27]. However, the prevalence of smear positive TB in this study was lower than the prevalence in the previous reports in Pakistan, 28.3% [28], Rwanda, 17.3% [29], Zambia, 28% [30] and in Ethiopia: Nekemit referral hospital 9.41% [31], Bale Goba and Robe hospitals 9.2% [32] and Agaro teaching health center, 10.9% [33]. This difference might be due to differences in the study populations as the previous studies were conducted among clinically TB suspected patients at public health care institutions. Patients usually visit the public health care facilities after they tried other treatment options.

In this study, the total family size per household was found to be statistically significant with smear positive pulmonary tuberculosis. This finding was consistent with cross sectional institution based studies in Bale Goba and Robe hospitals [32], and the Methara sugar factory hospital [34]. This might be due to the fact that poverty, malnutrition and over-crowded living conditions increase the risk of TB transmission [6, 35]. Since the transmission of tuberculosis is mainly indoor, living in large family size per household creates overcrowding conditions and increases the risk of acquiring tuberculosis. The other possible reason might be due to the fact that people with high number of family members tend to be of lower socioeconomic status.

In this study the prevalence of smear positive PTB was significantly associated with history of contact with active TB patents. Similar findings were reported from studies conducted in different part of Ethiopia [32, 36, 37]. Transmission of tuberculosis is mainly through aerosolized droplets and close contact with patients with active PTB facilitate acquisition of TB. If the person with tuberculosis is remaining active and untreated, it can have the possibility to infect 10 up to 15 people per year [38]. *Mycobacterium tuberculosis* is carried in airborne particles called droplet nuclei which are 1μm up to 5μm in diameter. Infectious droplet nuclei are generated from active tuberculosis patients during coughing, sneezing, speaking or singing. These small droplet nuclei can remain suspended in the air for several hours and transmitted to other nearby person through inhalation.

All smear positive PTB cases tested by GeneXpert MTB/RIF assay did not show resistance for RIF. Globally about 3.6% of new TB cases and 20.2% of previously treated cases had MDR-TB [3]. A study in Nigeria showed that previous history of anti-TB treatment was a significant factor for the development of drug resistance and MDR-TB [39]. In the present study 14 (3.7%) of the respondents were presented with history of previous anti-TB treatment. In addition to this, one of the smear positive cases was previously treated for PTB. In Amhara region, the prevalence of MDR-TB among new and retreated patients was 1.8% and 18.5%, respectively. Previous drug exposure was significantly associated with the development of drug resistance [40]. The reason for absence of RIF resistant cases in this study might be due to small number of smear positive PTB cases tested for rifampicin resistance. The presence of individuals with previous history of TB and history of anti-TB treatment in these study sites which is a major risk factor for the development of drug resistance could indicate the potential risk for transmission of MDR-TB among holy water users.

The limitation of this study was the use of direct smear microscopy alone for the diagnosis of TB. It may underestimate the prevalence of PTB in this study population. The use of advanced laboratory techniques like culture and molecular assays could give the best prevalence estimate of PTB among the study subjects.

## Conclusions

The prevalence of smear positive pulmonary tuberculosis in holy water sites was found to be 7.4 fold higher than the prevalence in the general population in Ethiopia. Previous history of contact with active tuberculosis patients and increased number of family members were identified as significant risk factors for smear positive pulmonary tuberculosis. Therefore, community health programs should consider holy water sites for health education, regular assessment of PTB suspects and refer to public health care institutions for early diagnosis and initiation of anti-TB treatment. Furthermore, large scale studies should be conducted using advanced laboratory techniques in holy water sites to better understand its impact for the transmission of TB, including MDR-TB, in the community.

## Abbreviations

HIV: Human immunodeficiency virus
MDR: Multidrug resistance
PTB: Pulmonary tuberculosis
RIF: Rifampicin
TB: Tuberculosis
